# Clinical recommendations for the inpatient management of lower respiratory tract infections in children and adolescents with severe neurological impairment in Germany—2026 Update

**DOI:** 10.1007/s00431-026-07482-5

**Published:** 2026-11-09

**Authors:** Maximilian David Mauritz, Ulrich von Both, Christian Dohna-Schwake, Christian Gille, Johannes Huebner, Markus Hufnagel, Johannes G. Liese, Hanna Renk, Henriette Rudolph, Ulf Schulze-Sturm, Florian Stehling, Tobias Tenenbaum, Boris Zernikow, Markus Knuf, Arne Simon

**Affiliations:** 1 Pediatric Palliative Care Center, Children’s and Adolescents’ Hospital, Datteln, 45711 Germany; 2 Department of Children’s Pain Therapy and Pediatric Palliative Care, Faculty of Health, School of Medicine, Witten/Herdecke University, Witten, 58448 Germany; 3 Department of Infectious Diseases, Dr von Hauner Children’s Hospital, LMU University Hospital, Munich, 80337 Germany; 4 Department of Pediatrics I, Neonatology, Pediatric Intensive Care Medicine, and Pediatric Neurology, University Hospital Essen, Essen, 45147 Germany; 5 Department of Neonatology, Heidelberg University Children’s Hospital, Heidelberg, 69120 Germany; 6 Department of General Paediatrics and Adolescent Medicine, Medical Faculty, University Medical Centre, University of Freiburg, Freiburg, 79106 Germany; 7 Department of Paediatrics, Division of Paediatric Infectious Diseases, University Hospital of Wuerzburg, 97080 Würzburg, Germany; 8 University Children’s Hospital Tuebingen, Tuebingen, 72076 Germany; 9 Department of Pediatrics, Goethe University Frankfurt, Frankfurt Am Main, 60590 Germany; 10 University Children’s Hospital, University Medical Centre Hamburg, Eppendorf, Hamburg, 20246 Germany; 11 Department of Pediatric Pulmonology and Sleep Medicine, University Children’s Hospital Essen, Essen, 45147 Germany; 12 Clinic for Child and Adolescent Medicine, Sana Klinikum Lichtenberg, Academic Teaching Hospital, Charité-Universitätsmedizin, Berlin, 10365 Germany; 13 Department for Pediatric and Adolescent Medicine, Worms Clinic, Worms, 67550 Germany; 14 Pediatric Oncology and Hematology, University Hospital Homburg Saar, Homburg/Saar, 66421 Germany

**Keywords:** Severe neurological impairment, Lower respiratory tract infections, Antibiotic treatment, Antimicrobial stewardship

## Abstract

Children and adolescents with severe neurological impairment (SNI) require specialized care due to their complex medical needs. These patients often experience severe, recurrent lower respiratory tract infections (LRTIs). These infections, of both viral and bacterial etiology, pose a significant risk to these patients, often resulting in respiratory insufficiency and long-term impairments. Based on expert consensus and newly available data, we have updated the previous 2024 clinical recommendations on the management of LRTIs in children and adolescents with SNI in Germany. This update now also addresses multidrug-resistant organism colonization, respiratory support, discharge planning, and prophylactic strategies. The recommendations emphasize comprehensive multidisciplinary care, antimicrobial stewardship, and a stepwise, risk-stratified approach to antibiotic therapy. Initial treatment should involve symptomatic care, including hydration, antipyretics, oxygen therapy, secretion management, and respiratory as well as cough support. In suspected bacterial LRTIs, antibiotic treatment (ABT) should be guided by clinical severity, previous microbiological findings, and treatment response, with an aminopenicillin plus a beta-lactamase inhibitor recommended for community-acquired LRTIs and piperacillin-tazobactam reserved for selected high-risk patients. Ongoing management includes regular reassessment, adjustment of ABT based on pathogen identification, and optimization of supportive care.

*Conclusion*: The updated recommendations provide a practical, multidisciplinary framework for the inpatient management of LRTIs in children and adolescents with SNI, combining optimized supportive care with antimicrobial stewardship and a stepwise, risk-stratified approach to antibiotic treatment.

**Table:** 

**What is Known:** •* Children and adolescents with severe neurological impairment are particularly affected by severe and recurrent lower respiratory tract infections (LRTI)* • *The indication and choice of antibiotic therapy for bacterial LRTI is often difficult because there are no evidence-based treatment recommendations for this heterogeneous but vulnerable patient population* • *The frequent overuse of broad-spectrum or reserve antibiotics in this patient population increases selection pressure for multidrug-resistant pathogens*	
**What is New:** *• Compared with the 2024 recommendations, this update provides more detailed guidance on secretion management and respiratory and cough support, introduces explicit discharge criteria, and expands decision-making for patients colonized with multidrug-resistant organisms. The antibiotic strategy has been further specified regarding broad-spectrum antibiotic therapy, while preventive recommendations have been expanded and updated.*

**Supplementary Information:**

The online version contains supplementary material available at https://doi.org/10.1007/s00431-026-07482-5.

## Introduction

Children and adolescents with severe neurological impairment (SNI) frequently have complex and distinctivemedical needs that require specialized care. SNI refers to various conditions marked by significant impairments in neurological functions such as motor skills, sensory processing, and cognitive abilities. These individuals often encounter significant challenges in everyday life and depend on comprehensive medical support to address their specific needs [1]. Other publications also describe these patients as “children with medical complexity” [2], “patients with neuromuscular diseases/disorders” [3, 4], “children with severe global developmental delay” [5], or “children with neurodisability” [6]. However, the SNI definition most accurately characterizes the patient group targeted by this treatment recommendation. Managing the medical needs of children and adolescents with SNI is a multifaceted task requiring a multidisciplinary approach. These individuals may experience difficulties across multiple health domains, including respiratory function, mobility, nutrition, and communication. Their conditions can result in limitations in muscle control, impaired sensation, and cognitive impairments, leading to a high risk of secondary health complications. Notably, these patients are affected by recurrent, sometimes severe, lower respiratory tract infections (LRTI) [7, 8]. The causes for the increased susceptibility to LRTI are complex and multifactorial. Risk factors for LRTIs include impaired secretion clearance (lower cough stimulus, ineffective cough), restrictive lung function, development of dys- and atelectasis, and micro- and macroaspiration due to dysphagia, seizures, or gastroesophageal reflux [9–12]. Polypharmacy in this patient group can also influence bone marrow function and lead to immunological side effects, e.g., due to secondary effects of antiepileptic drugs. Some of these patients are fitted with a tracheostomy or a gastrostomy, and on rare occasions, with a suprapubic bladder catheter or jejunostomy. For various reasons, some patients require respiratory support, including continuous positive airway pressure, non-invasive ventilation, and invasive ventilation via tracheostomy [13, 14]. Frequent nursing care interactions, emergencies, and autoinoculation (e.g., by aspiration) also increase the risk of transmitting pathogenic bacteria and viruses.

In patients with SNI, LRTIs account for a significant proportion of morbidity and are associated with an increased risk of death compared to healthy children [15, 16]. In addition, increased rates of LRTI-associated complications are to be expected in patients with SNI [17]. Recurrent LRTIs can lead to progressive respiratory insufficiency and, subsequently, affect the quality of life of these children, adolescents, and their families [18, 19]. A unique aspect of treating LRTI in these patients is the difficulty in obtaining representative microbiological samples from the lower airways [20]. In many cases, this is due to the difficulty of obtaining induced sputum from these patients. Contamination of airway samples by bacteria colonizing the upper respiratory tract is also common. Furthermore, viral LRTIs often present with a higher clinical severity in this patient group than in pediatric patients without SNI [21–24]and are often not clearly distinguishable from bacterial LRTIs [25].

In addition, the interpretability of conventional chest radiographs may be limited by factors such as frequent dystelectasis, scoliosis, and chronic pulmonary changes, as well as inadequate inspiratory depth and suboptimal positioning due to disease-related non-compliance with verbal instructions. Impaired secretion management in these patients promotes bacterial overgrowth. Many pathogens can form biofilms, for example, on an inserted tracheal cannula. In addition, patients with dysphagia who are on proton pump inhibitor therapy are at risk of pulmonary complications due to (micro-)aspiration of bacteria colonizing the stomach [26, 27].

There is an increased prevalence of multidrug-resistant (MDR) bacteria colonizing pediatric patients with SNI [28, 29]. This colonization often causes uncertainty about whether a different antibiotic therapy (ABT) is required for LRTI treatment [30, 31]. The excessive use of broad-spectrum or reserve antibiotics increases the selection pressure on MDR and is associated with acute adverse effects [32]. The resulting dysbiosis is more likely to pave the way for subsequent LRTIs, creating a vicious circle and constantly increasing the complexity of treatment strategies.

Patients with a tracheostomy are frequently and chronically colonized with MDR bacteria. Colonizing MDR bacteria can cause major problems in this context. However, not all patients with tracheostomy experience frequent LRTI. This complication is more likely to affect those with significant problems mobilizing respiratory secretions. Persistent airway secretions promote persistent (endobronchial) neutrophilic inflammation, ultimately leading to structural lung disease of the lower airways (e.g., bronchiectasis, chronic atelectasis) [33].

Since the initial publication of this guideline in 2024 [34], additional scientific societies in other countries have published recommendations for this population [35–37]. In addition, a first systematic review has summarized the available evidence on ABT of LRTI in children with SNI [6]. However, the included studies were largely retrospective and heterogeneous, and high-quality prospective evidence, particularly randomized evidence, remains very limited. Consequently, many aspects of diagnosis and treatment, including the 5 Ds of antimicrobial stewardship (AMS): diagnosis, antibiotic selection (drug), appropriate dose, treatment duration, and de-escalation strategies [38], remain insufficiently defined, contributing to considerable variability in clinical practice. This updated recommendation incorporates the available systematic evidence and aims to promote antimicrobial stewardship strategies that emphasize targeted, rather than unnecessarily broad-spectrum, ABT with appropriate treatment duration in this patient group.

## Materials and methods

These recommendations were prepared on behalf of the German Society for Pediatric Infectious Diseases (Deutsche Gesellschaft für pädiatrische Infektiologie, DGPI) and developed according to the regulations of the Working group of the German Scientific Medical Societies (Arbeitsgemeinschaft der Wissenschaftlichen Medizinischen Fachgesellschaften, AWMF). Professional societies were invited to participate based on the guideline’s topic and the aforementioned regulations [39]. Six professional societies agreed to participate in developing the clinical recommendations. Each participating society nominated one individual with specialized expertise in the topic: DGKJ (German Society for Pediatrics and Adolescent Medicine), represented by U.v.B.; DGPI (German Society for Pediatric Infectious Diseases), represented by T.T.; DGP (German Society for Palliative Medicine), represented by B.Z.; GNP (Society for Neuropediatrics), represented by M.K.; GNPI (Society for Neonatology and Pediatric Intensive Care Medicine), represented by C.D.-S.; and GPP (Society for Pediatric Pneumology), represented by F.S. The literature search was updated in August 2025 using Pubmed/MEDLINE and included publications from January 1, 2022, through August 2025 to ensure that studies published during the development of the original recommendations were also captured. The search terms were “pneumonia” or “lower respiratory tract infection” AND “neurological impairment” or “neuromuscular disease” or “neurodegenerative diseases” or “developmental disabilities” or “learning disability” or “medical complexity” or “neurodisability” or “developmental delay” AND “child*” or “pediatric*” or “infant*” or “adolescent*”. Titles and abstracts were screened for relevance to the inpatient management of LRTI in children and adolescents with SNI. Publications were considered relevant if they addressed diagnosis, antimicrobial treatment, secretion management, respiratory support, discharge planning, prevention, or other aspects directly relevant to the clinical management of this population or closely related pediatric populations.

No formal Grading of Recommendations Assessment, Development and Evaluation (GRADE) or other structured certainty-of-evidence assessment was performed, and evidence certainty and recommendation strength were not formally graded. The available evidence was sparse and heterogeneous and predominantly derived from observational studies, with only very limited randomized evidence. We therefore performed a narrative appraisal focusing on relevance to the target population, clinical applicability, consistency with the available literature, and antimicrobial stewardship implications. Proposed changes to the 2024 recommendations were based on new evidence or guidance supporting modification or expansion of the previous recommendations, or on areas in which the authors considered additional clinical guidance necessary.

An initial draft of the updated recommendations was discussed with the expert group during an online conference in December 2025. The group discussed all proposed changes until consensus was reached. A revised draft was subsequently circulated to the entire expert group for final review and confirmation of consensus. The manuscript was then submitted to the participating professional societies for review and approval. Following this process, all members of the expert group approved the final version. The German version of the recommendations was published in the AWMF guideline register [40]in March 2026. In July 2026, we made final adjustments in accordance with the revised national recommendations for pneumococcal vaccination [41]and the dosing guidelines for high-dose piperacillin-tazobactam, amoxicillin and fosfomycin [42, 43]. All members of the expert group reported potential conflicts of interest.

## Results

### Target population and scope of the recommendations

This treatment recommendation primarily covers the therapy of children with SNI admitted to hospitals as well as in inpatient long-term care facilities, including residential facilities for long-term ventilator-dependent patients, or children’s hospices with specialist care. The need for inpatient hospital treatment for these patients must be considered on an individual basis. The criteria for severe pneumonia, such as severe dyspnea, dehydration, prolonged capillary refill time, cyanosis, apnea, or impaired consciousness, are indicative [44]. Recommendations are made for pediatric patients with motor and/or cognitive impairments, and whose medical complexity requires interdisciplinary and multimodal support in activities of daily living [1], such as patients with severe cerebral palsy, neurodegenerative diseases, encephalopathies, syndromic diseases, progressive muscular or metabolic diseases, severe traumatic brain injury, or unfavorable courses of cerebral tumor disease.

### Diagnostics

Clinical signs of LRTI in these patients may include tachy- or dyspnea, fever, cough, increased thick, viscous, and discolored respiratory secretions, new onset or increased oxygen demand, or new pathologic auscultation findings. However, nonspecific signs, such as gastrointestinal symptoms, weight loss, increased restlessness, or decreased exercise tolerance, may also occur. Therefore, a thorough diagnostic workup is essential in this subset of patients. In patients who present as acutely unwell, timely recognition of septic shock and other sepsis-associated organ dysfunction and adequate treatment should be provided [45].

A detailed history and physical examination should be performed when patients are admitted to the hospital. In addition, vital signs such as temperature, respiratory rate, transcutaneous oxygen saturation, blood pressure, and diuresis should be obtained. The laboratory examination should include blood count, creatinine, C-reactive protein or procalcitonin, and blood gas analysis—including sodium, potassium, and calcium. In severe disease with clinical sepsis, a blood culture set should also be taken (aerobic and anaerobic; ideally directly during aseptic placement of the indwelling venous cannula). In children with long-term central venous access devices or catheters, both aerobic and anaerobic blood cultures should be obtained from the respective device/catheter.

For microbiological diagnostics, deep respiratory tract secretions should be obtained whenever possible and examined for respiratory bacterial pathogens. Ideally, the collection of secretions can be done by sampling sputum or nasopharyngeal aspirates after inhalation of hypertonic saline (e.g., 2–4 ml). During interpretation of these findings, it needs to be taken into account that these samples can contain commensal bacteria originating from the upper respiratory tract, particularly the nasopharynx. The detection of *Candida* spp. usually indicates colonization. Tracheal secretions should be collected from tracheostomy with a sterile suction catheter. Detecting multidrug-resistant Gram-negative bacteria (MDR-GNB) or methicillin-resistant *Staphylococcus aureus* (MRSA) in throat swabs or nasopharyngeal secretions does not necessarily indicate that these bacteria are causative agents of LRTI. For differential diagnosis, throat swabs should be tested (e.g., multiplex polymerase chain reaction) for respiratory viruses, such as severe acute respiratory syndrome coronavirus type 2, respiratory syncytial virus, influenza A and B, human metapneumovirus, parainfluenza virus, adenovirus, and rhinovirus. If severe *Mycoplasma* pneumonia is suspected, *Mycoplasma* PCR should be performed before starting macrolides or doxycycline. A positive result does not prove *Mycoplasma* LRTI, but a negative result will most likely exclude respiratory *Mycoplasma*infection [46]. For inpatient admission, screening for MDR bacteria should be performed according to local hygiene guidelines (e.g., nasopharyngeal combination swab for MRSA, anal swab for MDR-GNB, swab of the gastrostomy or tracheostomy and entry sites of devices, if inflamed). MDR screening is performed primarily for hospital hygiene (infection prevention) reasons. MDR bacteria colonizing the child may not necessarily be the causative agent of a LRTI; positive findings (including previous findings) must be critically evaluated [47].

For further evaluation, ultrasonography of the thorax is recommended in cases of suspected pulmonary consolidation, particularly when pleural effusion or empyema is also suspected [48]. Not every patient with LRTI requires a chest X-ray (CXR). However, in cases of new-onset symptoms, worsening hypoxemia (or ventilatory dysfunction), or an unclear suspected diagnosis, a CXR should be considered. In patients requiring intensive care or those with recurrent LRTI or difficult-to-interpret conventional radiographic findings, computed tomography (CT) of the chest may be considered during the infection-free interval.

### Initial treatment

All patients should receive adequate symptomatic therapy with weight-adjusted hydration, antipyretic therapy for persistent fever, respiratory support and oxygen therapy if transcutaneous saturation is less than 91%, as well as inhaled bronchodilator and secretolytic therapy if needed. Supportive respiratory physiotherapy should be provided to patients with retained airway secretions. In case of severe disease with respiratory insufficiency (oxygen demand, hypercapnia), the responsible pediatric pneumologists, infectious diseases specialists, and intensive care specialists should be consulted at an early stage (e.g., indication for intensification of an established respiratory therapy or the start of non-invasive or invasive ventilation, high-flow respiratory support, or cough assistant). Severe dyspnea (complaints from the patient or immediate caregiver should guide treatment decisions) can be treated pharmacologically, for instance, with regular or continuous administration of strong opioids. Drug administration can be either oral (or via gastric tube) or intravenous. In the case of dyspnea attacks, patients can be treated with weight-adjusted intranasal fentanyl [49, 50].

If there is no clinical response to ABT, there should be a discussion before switching (or escalating) ABT on whether the lack of clinical response is attributable to other reasons (notably inadequate secretion management), so that an improvement in symptomatic treatment can alleviate the patient's clinical state independently of ABT.

### Suspected viral LRTI

If influenza is strongly suspected (e.g., influenza is detected in a symptomatic contact person) or influenza viruses are detected in respiratory secretions, a 5-day course of oral (or gastric tube) oseltamivir should be started. Given the short time window for effective treatment with oseltamivir, it should be started as soon as possible. Although the real benefits and adverse consequences of oseltamivir treatment are debated, this expert group recommends oseltamivir given the potential long-term pulmonary sequelae of influenza infection in this patient group [51, 52]. In case of clinical deterioration, re-evaluation for bacterial coinfection should be performed after 48–72 h. In patients with, or immediately after, influenza infection, pneumococci and*S. aureus*are the most common pathogens of bacterial coinfection [53].

### Suspected bacterial LRTI

The initial ABT should be tailored based on the patient’s group classification. Patients with community-acquired LRTI or suspected aspiration pneumonia are categorized as group I and should receive an aminopenicillin combined with a beta-lactamase inhibitor. This approach effectively covers the most common pathogens responsible for childhood community-acquired pneumonia, such as pneumococci, *Haemophilus influenzae, Moraxella catarrhalis*, group A streptococci, and methicillin-susceptible *S. aureus*, excluding *Mycoplasma*spp. [54, 55]. This combination also covers many anaerobic species relevant to these patients. It is also effective against pneumococci with reduced penicillin susceptibility (excluding resistant isolates) when high-dose aminopenicillins are used (150 mg/kg/day ampicillin intravenously or 90 mg/kg/day amoxicillin orally) [56]. Depending on the severity of the LRTI, initial therapy may also consider known colonization or experience with previous episodes of hospitalized LRTI [57]. Group II patients are those with chronic lung disease and/or with tracheostomy and frequent recurrent LRTI. Keeping in mind that for some of these patients this may be too broad in spectrum, initial calculated therapy with piperacillin-tazobactam is recommended until the results of the microbiological diagnostics become available. The extension to piperacillin-tazobactam primarily means an increase in efficacy against Gram-negative bacteria (GNB), including isolates with beta-lactamases inhibited by tazobactam and*P. aeruginosa.* This pragmatic risk stratification should not lead to liberal use of piperacillin-tazobactam. The benefits of broader Gram-negative coverage, including activity against *P. aeruginosa*, concerning clinical and microbiological healing rates remain uncertain; some cohort studies in patients with neuromuscular disorders reported infrequent detection of *P. aeruginosa*[6, 58]. In non-critically ill patients without prior isolation of*P. aeruginosa* from respiratory specimens, initial ampicillin-sulbactam therapy for 48 h appears reasonable. The individual medical history, including pretreatment with broad-spectrum antibiotics and microbial findings during earlier events, should be considered. Patients with chronic or recurrent *P. aeruginosa*airway colonization should be treated according to principles for non-CF bronchiectasis [6]; in clinically stable patients with documented susceptibility, oral ciprofloxacin may be considered as primary or step-down therapy. Table1 summarizes specific treatment recommendations.

**Table 1:** Empiric antibiotic therapy for suspected bacterial LRTI

Patient group	Therapy recommendation
Group I: Community-acquired LRTI or aspiration pneumonia^#^	Ampicillin-sulbactam intravenously150 mg/kg/day related to the ampicillin fractiondivided in 3 doses (maximum single dose 2/1 g) *or*
	Amoxicillin-clavulanic acid 7:1 orally or by gastric tube(50-)90* mg/kg/day related to the ampicillin fractiondivided in 3 doses (maximum single dose 875 mg amoxicillin)* or*
	Sultamicillin orally or by gastric tube(50-)90* mg/kg/day related to the ampicillin fraction divided in 3 doses (maximum single dose 750 mg sultamicillin).
Group II: Patients with chronic lung disease and/or with tracheostomy and frequent recurrent LRTI^#^	Piperacillin-tazobactam intravenously400 mg/kg/day** related to the piperacillin fraction divided in 4 doses (maximum single dose 4/0.5 g).

*LRTI* lower respiratory tract infection. * High-dose amoxicillin/ampicillin (90 mg/kg/day) is supposed to be reserved for the treatment of pneumococcal infections with reduced penicillin susceptibility; this should be taken into account depending on the local epidemiological situation. ** High-dose piperacillin-tazobactam is recommended for Pseudomonas aeruginosa coverage. # Depending on the severity, taking into account known respiratory tract colonization or based on experience with previous episodes of treated LRTI in this patientCompared with the 2024 recommendations, aminopenicillin and piperacillin-tazobactam dosing were updated, and risk-based considerations for initial therapy were also added to the table (#)

**Fig. 1:**
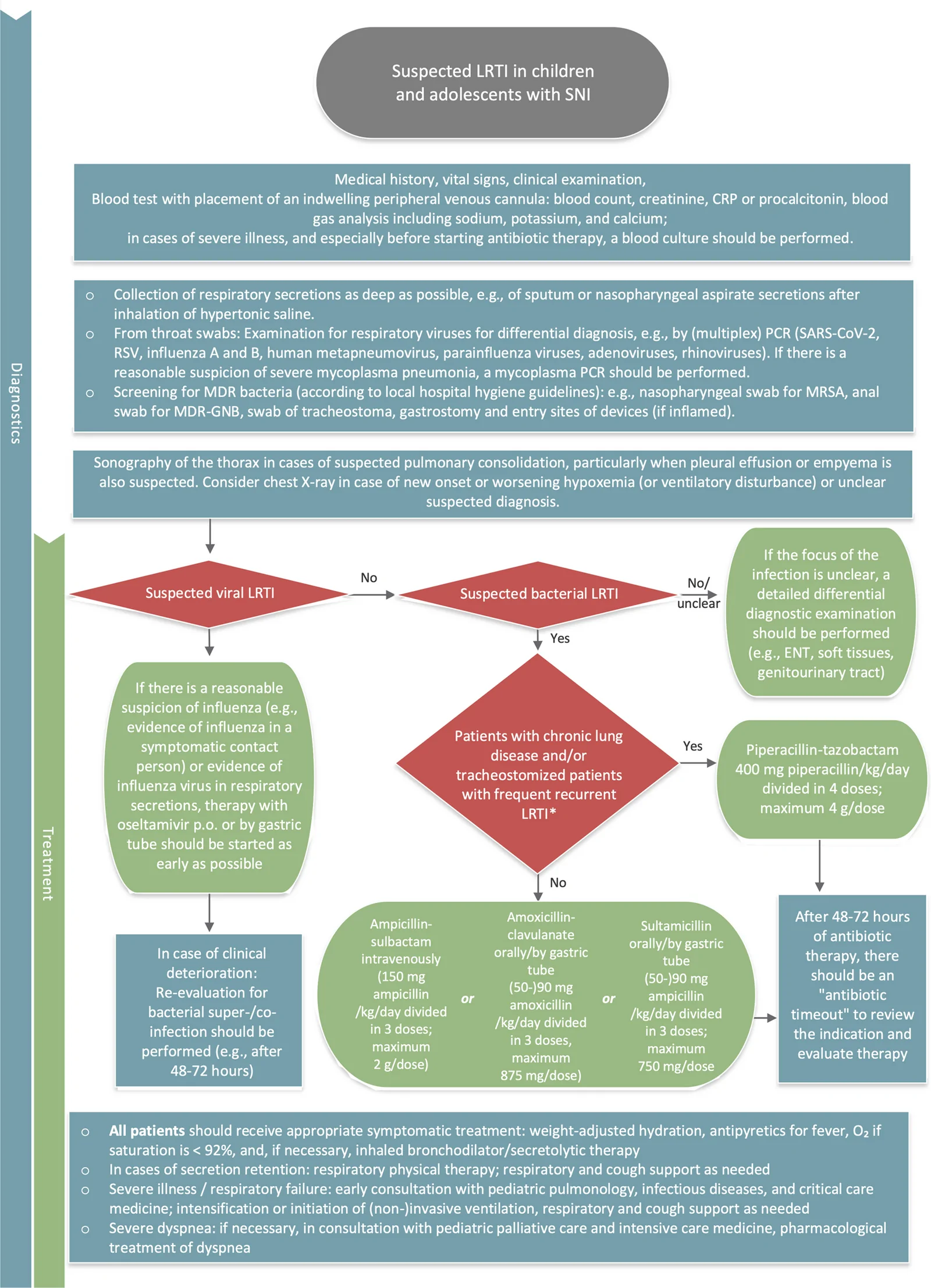
Initial diagnostic and therapeutic measures for lower respiratory tract infections in children and adolescents with severe neurologic impairment. Kg: kilogram, LRTI: lower respiratory tract infection, MRSA: methicillin-resistant *Staphylococcus aureus*, MDR: multidrug-resistant, PCR: polymerase chain reaction, RSV: respiratory syncytial virus, SARS-CoV-2: severe acute respiratory syndrome coronavirus type 2, SNI: severe neurological impairment. * Depending on the severity, taking into account known respiratory tract colonization or based on experience with previous episodes of treated LRTI in this patient. Compared with the 2024 version, antibiotic dosing was updated, and the supportive management section was expanded to provide more detailed guidance on secretion management and respiratory and cough support

All critically ill patients should be primarily treated with piperacillin-tazobactam, irrespective of group assignment. If LRTI is present with septic shock or sepsis with multiple organ failure, initial therapy should include meropenem plus vancomycin or teicoplanin, for example. Initial diagnostic and therapeutic measures are summarized in Fig. 1.

In patients with SNI and LRTI who are colonized with MDR-GNB and are not critically ill, particularly when colonization is documented outside the respiratory tract, previous isolates should not automatically determine initial empiric therapy. Depending on disease severity and previous episodes of hospital-treated LRTI, prior microbiological findings may be considered. Adding fosfomycin or an aminoglycoside to ampicillin-sulbactam or piperacillin-tazobactam may bridge the interval until current susceptibility results are available and avoid early use of meropenem, ceftazidime-avibactam, ceftolozane-tazobactam, or tigecycline. Similarly, in non-critically ill patients colonized with MRSA, particularly outside the respiratory tract, empiric MRSA coverage is mandatory only if there is evidence of complicated LRTI, such as abscess formation or pleural empyema. If available, the susceptibility profile should be reviewed (e.g., testing susceptibility of linezolid and trimethoprim-sulfamethoxazole), as alternatives to vancomycin or teicoplanin may be appropriate. For targeted or step-down therapy, clindamycin, trimethoprim-sulfamethoxazole, doxycycline, or linezolid may be considered according to susceptibility results, with attention to pediatric dosing, contraindications, and drug interactions.

Critically ill patients colonized with MDR-GNB should receive initial empiric therapy guided by the most recent susceptibility profile. Critically ill patients colonized with MRSA should receive empiric MRSA coverage; if susceptibility results are unknown, linezolid (off-label), vancomycin, or teicoplanin may be used, with no clear preference among the expert group. Therapeutic drug monitoring is recommended to confirm an appropriate systemic exposure and to avoid toxicity when glycopeptides are used for severe infections, although the therapeutic targets (trough level, AUC/MIC) are not well defined in this specific population. In all patients receiving initial combination therapy, an antibiotic timeout after 48–72 h is essential, as treatment can often be narrowed to targeted monotherapy.

For patients with chronic multidrug-resistant organism (MDRO) colonization, an individualized anticipatory treatment plan should be developed together with the local AMS team and made readily available for future LRTI episodes.

### Secretion management and respiratory support

Children and adolescents with SNI frequently have impaired airway clearance due to oromotor dysfunction, bulbar respiratory impairment, ineffective cough, and muscle weakness. During LRTI, retained airway secretions increase the risk of atelectasis, respiratory decompensation, and bacterial superinfection. The effectiveness of secretion management should therefore be assessed in all patients with SNI and signs of LRTI. Clinical signs of increased secretion burden, such as coarse crackles, productive cough, atelectasis, increased oxygen, or ventilatory requirements, should prompt targeted respiratory physiotherapy, including manual airway clearance techniques and, in patients with known or suspected ineffective cough, mechanical insufflation-exsufflation (MI-E). Inhaled isotonic or hypertonic saline or bronchodilators may be used prior to airway clearance to facilitate secretion mobilization. Suctioning may be required in patients with dysphagia or inability to clear mobilized secretions.

Patients who already have airway clearance routines can maintain them as long as their work of breathing remains stable. If respiratory effort increases or secretion retention is suspected, treatment should be intensified, for example, by increasing the frequency of MI-E to at least 3 times daily, increasing the applied pressures or by adapting the technique. In patients without prior airway clearance support, measures should be initiated during an acute pulmonary exacerbation if cough is ineffective, secretion burden is significant, expectoration is difficult, or atelectasis persists. All airway clearance interventions should be coordinated with the local pediatric respiratory, intensive care, and respiratory therapy team, and gradually reduced once the secretion burden and clinical status improve.

**Fig. 2:**
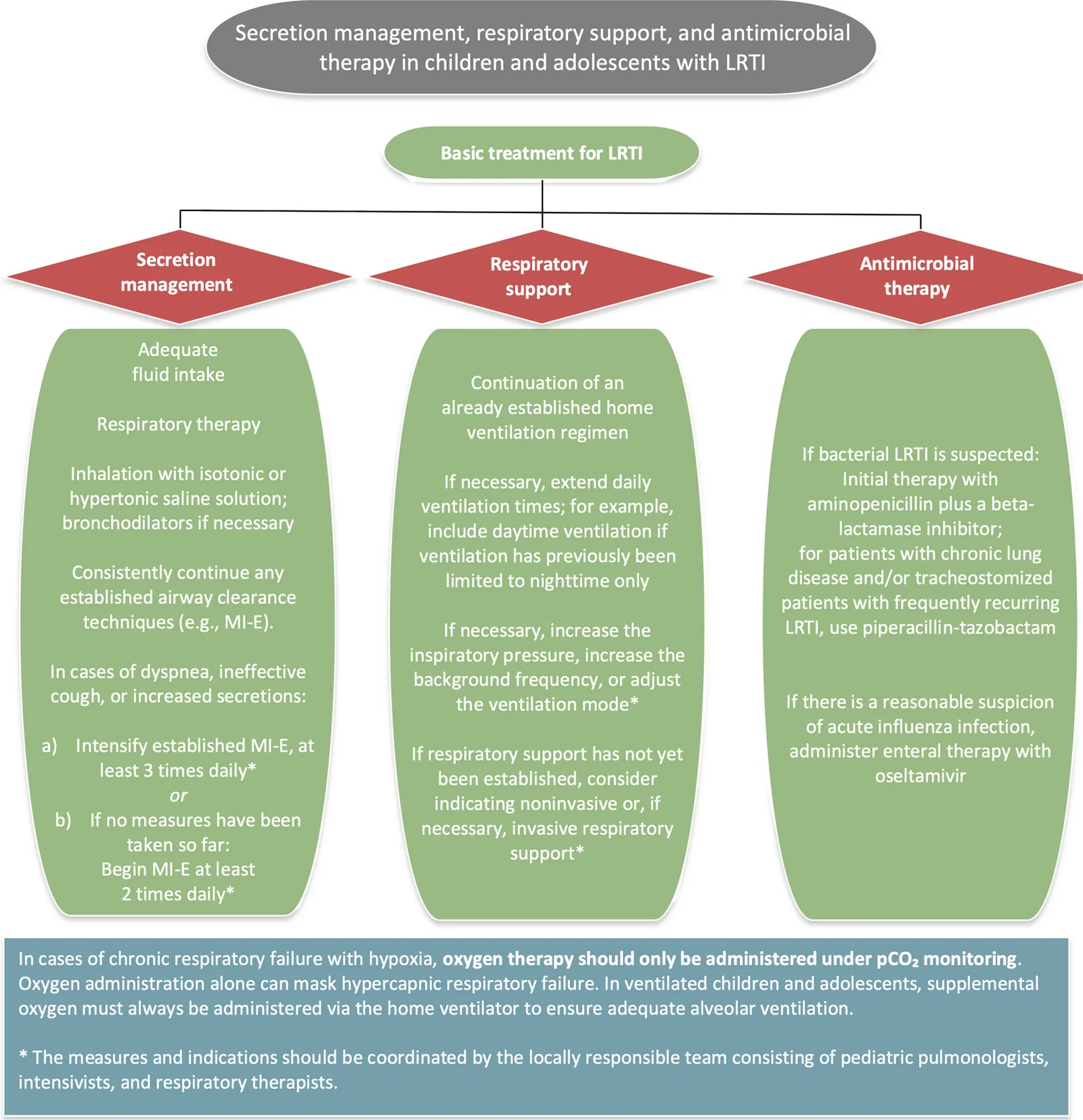
Supportive measures for secretion management and respiratory support in addition to antimicrobial therapy. pCO2: partial pressure of carbon dioxide. MI-E: mechanical insufflation-exsufflation. This figure was newly added in the 2026 update to summarize recommendations on secretion management and respiratory support

Children and adolescents with chronic respiratory insufficiency receiving nocturnal or continuous non-invasive or invasive ventilation should continue ventilatory support during acute LRTI. Signs of respiratory compromise, including tachypnoea, use of accessory muscles, decreasing oxygen saturation, or reduced alertness, should prompt reassessment of ventilator settings and oxygen requirements. Temporary escalation may include longer ventilation periods, daytime ventilation, or adjustment of inspiratory pressure, backup rate, or ventilation mode. Oxygen therapy should be administered under controlled conditions with monitoring of ventilation, including pCO₂, as oxygen supplementation may mask hypercapnic respiratory failure. If additional oxygen is required in children receiving home ventilation, it should be delivered via the ventilator to ensure adequate alveolar ventilation. Secretion management and respiratory support measurements are summarized in Fig. 2.

### Ongoing management

After 48–72 h of antibiotic therapy, an “antibiotic timeout” should be performed to review the indication and evaluate the current therapy. This timeout should be planned when the patient is admitted, preferably with an infectious disease consultation. All available information should be collected and reviewed to assess the likelihood of a bacterial infection in the patient. The clinical course under antibiotic therapy, X-ray/CT findings, laboratory findings, and the microbiological results of bacterial and virological diagnostics should be taken into consideration.

### If the patient’s clinical condition improves

If bacterial infection is considered unlikely during the “antibiotic timeout,” ABT should be discontinued. If a bacterial infection is suspected and the patient’s condition improves with the initiated therapy, the ABT should be adapted, where feasible, to target the specific pathogen that may have been detected (e.g., penicillin, ampicillin, or amoxicillin instead of ampicillin-sulbactam). Importantly, a switch from intravenous to oral ABT (or, via a gastric tube) should be considered whenever possible.

### If the patient’s clinical condition remains poor

If the patient’s condition remains persistently poor, all aspects of supportive care need to be reviewed (e.g., inhalation, secretolysis, physical therapy, positioning, respiratory and cough support, dyspnea management, and pain management). If there are new relevant findings upon clinical examination or imaging, alternative diagnoses or complications should be considered. After infectious diseases and/or pulmonary consultation, a repeat CXR or ultrasound can determine the presence of pleural empyema. Bronchoalveolar lavage may be necessary. If pathogens are detected and the resistogram results are available, a targeted change of ABT should be performed. For LRTI due to *Pseudomonas* spp., piperacillin-tazobactam should be administered every 6 h. In addition, if vascular access permits, prolonged infusion administration over 3–4 h is a valuable option to improve ABT.

### If the patient’s clinical condition worsens significantly

If the patient’s condition worsens significantly during therapy, all aspects of supportive care (e.g., inhalation, secretolysis, physical therapy, positioning, respiratory and cough support, dyspnea management, and pain management) should be optimized first. In the case of pathogen detection from respiratory specimens and available resistogram results, a targeted change of ABT should be performed. If no pathogens are detected, therapy should be escalated. In group I patients treated with an aminopenicillin plus a beta-lactamase inhibitor, therapy should be switched to piperacillin-tazobactam.

The various options for escalating therapy in patients previously treated with piperacillin-tazobactam (group II) should be adapted to the individual patient [6]. For example, patients with severe peripheral neuropathy, pre-existing renal dysfunction, or sensorineural hearing loss should not receive aminoglycosides. In this case, fosfomycin or aztreonam are possible alternatives. The alternatives listed in Table 2 are intended to promote a generally restrictive and, where necessary, well-justified use of carbapenems.

**Table 2:** Possible escalation of antibiotic therapy (in the absence of pathogen identification)

Patient group	Therapy recommendation
Group I: Escalation of aminopenicillin plus beta-lactamase inhibitor to:	Piperacillin-tazobactam intravenously400 mg/kg/day** related to the piperacillin fractiondivided in 4 doses (maximum single dose 4/0.5 g), subsequent re-evaluation after 48–72 hours
Group II: Escalation of piperacillin-tazobactam (alphabetical):	Combination of piperacillin-tazobactam plus intravenous tobramycin* 7.5 mg/kg/day once daily *or*
	Combination of piperacillin-tazobactam plus fosfomycin intravenously 300 mg/kg/day divided in 3 doses(maximum single dose 8 g) *or*
	Switch to cefepime intravenously 150 mg/kg/day divided in 3 doses, extend infusion duration to 2–4 hours from second administration (maximum single dose 2 g)plus fosfomycin intravenously 300 mg/kg/day divided in 3 doses, (maximum single dose 8 g) *or*
	Switch to levofloxacin intravenously or oral/by gastric tube (infusion for min 1 h), < 5 years: 20 mg/kg/day divided in 2 doses; > 5 years: 10 mg/kg/day once daily (maximum single dose 750 mg) *or*
	Switch to meropenem intravenously 60 mg/kgKG/day divided in 3 doses (maximum single dose 1 g)

Compared with the 2024 recommendations, dosing was updated, including high-dose piperacillin-tazobactam and revised fosfomycin dosing, and the escalation options were streamlined by removing the ceftazidime–tobramycin combination

*LRTI* lower respiratory tract infection. *Potentially nephro- and ototoxic; therapeutic drug monitoring is recommended to document adequate elimination. ** High-dose piperacillin-tazobactam is recommended for Pseudomonas aeruginosa coverage

Ciprofloxacin is the preferred fluoroquinolone for infections caused by *P. aeruginosa*[59]. Levofloxacin may be considered only in selected cases requiring empiric oral coverage when no causative pathogen has been identified. Fluoroquinolones in children should generally be used with caution due to the risk of long-term selection of MDR bacteria and potential neurotoxicity. Including fluoroquinolones in this list should not be misinterpreted as a “license for uncritical use,” especially in children and adolescents with SNI who are treated as outpatients.

### Duration of antimicrobial therapy, discharge criteria

Antibiotic therapy, with clinical efficacy usually seen after 48–72 h, should usually be given for (5-)7 days [6]. The shorter duration may be sufficient in selected cases with rapid clinical improvement. A longer treatment duration is required for complicated LRTI, such as parapneumonic effusion or pleural empyema, or as determined by clinical response. For patients with bronchiectasis, a 14-day treatment duration is recommended unless a favorable clinical course suggests a shorter duration. Sequential oral therapy is possible according to susceptibility results. Suitable agents for oral step-down therapy may include amoxicillin-clavulanate, sultamicillin, cephalexin, and clindamycin, as well as trimethoprim-sulfamethoxazole and doxycycline (in children aged 8 years and older). Treatment should be guided by the susceptibility profile whenever available; otherwise, the most recently effective antibiotic regimen should be considered.

Children and adolescents who require intensified respiratory or cough support during treatment may be considered medically fit for discharge once support has been de-escalated, general condition is stable, and pulse oximetry has returned to the patient’s individual baseline. Discharge with moderately intensified respiratory or cough support may be appropriate if the patient’s condition has improved, oxygenation has returned to baseline, support parameters are safe for outpatient care, and short-term follow-up is scheduled to guide further de-escalation. Discharge documents, including the discharge letter and ventilation prescription, should include a plan for de-escalation or weaning of respiratory and cough support.

### Prevention of (recurrent) LRTIs and considerations for future treatments

Non-pharmacological prevention includes standard hygiene measures; infection prevention in children with devices such as tracheostomies, percutaneous endoscopic gastrostomies, or central venous access; optimization of ventilator settings to prevent atelectasis and secretion retention; treatment of gastro-oesophageal reflux; and adequate seizure control. In particular, good oral hygiene, including daily dental care and regular dental review twice yearly, has been associated with the prevention of aspiration pneumonia [7, 60].

In patients with SNI and recurrent bacterial LRTIs caused by GNB, inhaled antibiotics such as tobramycin or colistin may be considered, depending on susceptibility results, for prophylaxis or pathogen eradication, analogous to treatment approaches in patients with cystic fibrosis, for example, in 28-day on/off cycles [61]. Reliable data on enteral antibiotic prophylaxis in patients with SNI are lacking. Previous antibiotic exposure may adversely affect the microbiome, promoting dysbiosis and potentially increasing the risk of subsequent infections [7]. Long-term anti-inflammatory azithromycin should therefore be used only after careful individual risk–benefit assessment, as it selects for macrolide-resistant bacteria and may contribute to co-selection of penicillin-resistant pneumococci [62, 63].

Vaccination against the most common bacterial and viral LRTI pathogens is important to reduce antibiotic overuse and the subsequent development of antibiotic resistance. For pneumococcal immunization, children and adolescents with SNI aged 2–17 years should receive a 20-valent pneumococcal conjugate vaccine (PCV) according to current recommendations; if PCV13 or PCV15 vaccine has previously been administered, PCV20 vaccine should be given 1 year after the last conjugate vaccine dose [41]. In addition, passive RSV and active seasonal immunizations against influenza and COVID-19 should be administered in accordance with current national recommendations.

Analogous to the procedure for CF patients, a strategy for future empiric ABT should be defined for children and adolescents with SNI and recurrent bacterial LRTI. This plan should be accessible to all medical personnel involved in their care, such as including it in the patient’s file note or as a recommendation in their hospital discharge letter. For patients with recurrent bacterial LRTIs for whom oral medications have not been effective in previous LRTIs, early implantation of a long-term central venous access device can help both the patients and the medical staff involved.

### Ethical and palliative care aspects

The medical treatment of children and adolescents with severe neurological disorders, regardless of acute LRTI, has the overarching goal of maximizing quality of life until the end of life. Children and adolescents with SNI often have a limited life expectancy and are burdened by various symptoms with limited causal therapeutic treatment options. Depending on the complexity of the disease, specialized palliative medical co-treatment should be considered.

Before implementing any therapeutic measure, both a medical indication and the patient’s (presumed) will are required. In situations involving life-threatening crises and infections in the context of the SNI-related illnesses described above, palliative and symptom-relieving therapy may take precedence during the treatment over curative goals focused on healing and prolonging life. Individual therapy goals and decisions should be made through specialist consultation involving participatory decision-making, either with patients capable of giving consent or with their legal guardians or caregivers. The local clinical ethics committee, palliative physicians, hospital chaplains, or psychologists can provide support during this process. Regardless of the goal of the therapy, the aim is always to alleviate distressing symptoms such as pain, shortness of breath, anxiety, or restlessness, and to provide holistic, family-centered support.

In patients with life-limiting diseases, there may be instances where certain burdensome, life-prolonging interventions are no longer desirable, with a greater emphasis placed on specialized palliative care. Therapeutic limitations should be considered when clinical experts determine that implementing new interventions such as intensive therapy escalation (e.g., ventilation, central catheter placement) are highly likely to only prolong the dying phase or otherwise excessively burden the patient rather than overcome acute medical deterioration. Advance care planning (ACP) at times when patients are not in a critical phase of life helps glean the wishes of patients and their guardians for the different phases of disease deterioration. ACP should be conducted for all patients with SNI [64]. Part of this process can be an “Advance declaration on emergency situations,” which predefines specific treatment preferences to be communicated by the aforementioned caregivers or patients capable of giving consent in the event of deterioration in the underlying disease or associated complications (e.g., severe or recurrent LRTI). The existence of such an advance directive does not absolve the treatment provider of their obligation to coordinate the current treatment as early as possible with representatives, and if necessary, with patients capable of giving consent. The expression of the patient’s will by the patient capable of giving consent, or by the patient’s legal guardians or caregivers, always takes precedence over any advance directive.

## Summary of recommendations

The following section summarizes the key clinical recommendations, which are also provided in structured form in the supplementary material 1. Managing the medical needs of children and adolescents with SNI is a multifaceted task that requires a comprehensive multidisciplinary approach. These recommendations aim to provide guidance for optimizing the diagnosis and treatment of LRTI in children and adolescents with SNI.

All children and adolescents with SNI and acute LRTI should receive adequate symptomatic therapy, including physiotherapy, positioning, dyspnea treatment, and pain therapy as needed. In patients with impaired airway clearance, secretion management and respiratory support should be assessed early and initiated or intensified if clinically indicated. Children and adolescents receiving non-invasive or invasive ventilation should continue ventilatory support during acute LRTI, with temporary adjustments to ventilator settings, ventilation times, and oxygen supplementation as needed. Patients should receive adequate medical and microbiological diagnostics in line with these recommendations, including a workup concerning possible differential diagnoses. When starting ABT, an “antibiotic timeout” after 48–72 h should be established to check the indication and evaluate the therapy in a multidisciplinary team of clinicians and infectious diseases specialists or an AMS team. In the case of suspected bacterial LRTI, a calculated antibiotic therapy with an aminopenicillin plus a beta-lactamase inhibitor should be given, and in patients with known chronic lung disease and/or with tracheostomy and frequently recurring LRTI, piperacillin/tazobactam is considered the drug of choice. Previous colonization with MDR-GNB or MRSA should not automatically warrant broad empiric treatment in non-critically ill patients, particularly if colonization was documented outside the respiratory tract; however, prior microbiological findings and prior LRTI episodes should be considered in relation to disease severity. In critically ill patients colonized with MDR-GNB or MRSA, initial empiric therapy should take the most recent susceptibility profile into account, followed by early narrowing whenever clinically appropriate. The indication for ABT should be reassessed throughout the treatment course during scheduled “antibiotic timeouts.” Detecting a respiratory virus infection can assist in the decision to discontinue an initiated ABT. However, clinical and laboratory findings should also be considered. Detecting a respiratory virus does not exclude the possibility of a bacterial (co-)infection [65, 66]. If a bacterial LRTI is likely, ABT should be adjusted based on relevant microbial findings from the respiratory specimen at this time. This should involve focusing ABT and can involve an intravenous-to-oral switch. Antibiotic therapy should usually be given for 7 days, with shorter treatment possible in selected patients with rapid improvement and longer treatment required in complicated LRTI or bronchiectasis. In the absence of improvement of the clinical condition, all elements of symptomatic therapy should first be optimized. In the case of significant deterioration, empirical escalation of therapy should be initiated in the absence of evidence of a pathogen from respiratory material or blood cultures. Medical discharge may be considered once respiratory support has been de-escalated, clinical status is stable, and pulse oximetry has returned to the patient’s baseline; discharge with moderately intensified support may be appropriate if outpatient safety and short-term follow-up are ensured. Prevention of recurrent LRTI should include non-pharmacological measures, including oral/dental care and avoidance of routine long-term enteral antibiotic prophylaxis; selective use of inhaled antibiotics for recurrent Gram-negative LRTIs based on susceptibility results; and completion of immunization schedules in accordance with national recommendations, including RSV, COVID-19, influenza, and pneumococcal immunizations.

## Discussion

Compared with the 2024 recommendations, the present update retains the original stepwise antimicrobial stewardship framework but adds detailed guidance on secretion management, respiratory and cough support, explicit discharge criteria, and management of multidrug-resistant organism colonization. It also clarifies risk-stratified use of broad-spectrum and *Pseudomonas aeruginosa*-active ABT and updates preventive recommendations. These changes were informed by incorporating newly published international guidance [36, 37]and by systematic review data on ABT for LRTI in children with SNI [6]; when direct evidence remained limited, recommendations were based on expert consensus. Although the evidence base remains limited and largely retrospective, the available data support the central principle of these recommendations: Initiate ABT in a targeted, risk-stratified manner and escalate only in the event of defined treatment failure or in response to microbiological findings.

The rationale for the initial ABT proposed in these recommendations arose in particular from the transfer of experience in treating patients with CF non-CF bronchiectasis. As in this patient group, pediatric patients with SNI also harbor pathogens such as *Pseudomonas aeruginosa*, *Staphylococcus aureus*, and Enterobacteriaceae, which play an important role in the development of LRTIs, in addition to the typical pathogens of community-acquired pneumonia (CAP) in otherwise healthy children and adolescents [67–70]. Nevertheless, the more intensive (broader-acting and longer-lasting) ABTs used in patients with CF should not be applied blindly to all patients with SNI and LRTI, given the lack of high-quality prospective studies and the risk of long-term selection of MDR bacteria with a “one-size-fits-all” approach. The proposed empiric therapy options cover common bacterial pathogens of CAP in children [55],*S. aureus*, and many GNB [71, 72], as well as rare cases of anaerobic LRTIs [73]. If these therapies fail and the pathogen is undetected, we assume that the most likely cause is an insufficiently treated GNB. This stepwise approach is supported by a recent systematic review on ABT of LRTI in children with SNI, in which anaerobic, Gram-positive, and Gram-negative coverage was frequently adequate, whereas*P. aeruginosa*-active therapy appeared most relevant in patients who were more severely ill at presentation or failed to improve [6]. Although all included studies were of low quality, these findings support the concept of initiating ABT with an aminopenicillin plus a beta-lactamase inhibitor and subsequently escalating according to clinical severity and treatment response, rather than routine broad-spectrum therapy for all patients with SNI and LRTI.

Hence, this is the rationale for expanding the spectrum of activity by either switching from aminopenicillin plus beta-lactamase inhibitor to the broader Gram-negative and *P. aeruginosa*-active piperacillin-tazobactam or further expanding the Gram-negative spectrum of the existing piperacillin-tazobactam therapy. In this context, there is favorable evidence from CF therapy for combination therapy with a beta-lactam antibiotic or a *P. aeruginosa*-active cephalosporin [74, 75]and intravenous tobramycin [76, 77]or intravenous fosfomycin [78–80]. Extended infusion of beta-lactam antibiotics may be considered depending on the clinical situation and vascular access [81]. We propose levofloxacin as a second-line oral treatment option for patients for whom intravenous administration is not feasible or not desired due to individual circumstances. Levofloxacin has good bioavailability and broad-spectrum activity against most Gram-negative and Gram-positive bacteria [59]. All options are intended to provide an alternative treatment choice alongside carbapenems for non-critically ill patients. Inhaled ABT, especially with tobramycin, can support acute treatment [82–84].

In addition to established immunizations against common bacterial pathogens such as *Haemophilus influenzae* and *Streptococcus pneumoniae*, immunizations against viral pathogens can help prevent severe courses of LRTI. Implementation of these recommendations may improve diagnostics and targeted therapy for LRTI in these patients. In particular, the targeted use of antibiotics should prevent the development of resistance in colonizing bacteria.

### Limitations and critical research questions

Few studies exist on evidence-based prevention and treatment of lower respiratory tract infections in children and adolescents with severe neurological impairment. The available evidence remains sparse and heterogeneous and is predominantly derived from observational studies, with only very limited randomized evidence. In the systematic review by Marpole et al., the certainty of evidence for antibiotic treatment was rated as low to very low using GRADE [6]. Consequently, the present recommendations are consensus-based expert recommendations rather than formally evidence-graded recommendations. Research questions for future evidence-based recommendations include the following: Which pathogens cause LRTI in children and adolescents with SNI? Which interventions have a preventive effect on developing lower respiratory tract infections? What prophylactic therapies protect against recurrent lower respiratory tract infections (including consideration of resistance development)? What calculated antibiotic therapy and escalation strategy for children and adolescents with severe neurological impairment has a reasonable response rate without increased risk of resistance development of colonizing bacteria? What is the optimal length of therapy for LRTI in children and adolescents with SNI?

## Supplementary Information

Below is the link to the electronic supplementary material.

ESM 1 (DOCX 27.6 KB)

## Data Availability

No datasets were generated or analysed during the current study.

## Abbreviations

ABT: Antibiotic therapy
ACP: Advance care planning
AMS: Antimicrobial stewardship
CAP: Community-acquired pneumonia
CF: Cystic fibrosis
CT: Computed tomography
CXR: Chest X-ray
GNB: Gram-negative bacteria
GRADE: Grading of Recommendations Assessment, Development and Evaluation
Kg: Kilogram
LRTI: Lower respiratory tract infection
MRSA: Methicillin-resistant *Staphylococcus aureus*
MDR: Multidrug-resistant
MDR-GNB: Multidrug-resistant Gram-negative bacteria
MDRO: Multidrug-resistant organism
MI-E: Mechanical insufflation-exsufflation
PCR: Polymerase chain reaction
PCV: Pneumococcal conjugate vaccine
RSV: Respiratory syncytial virus
SARS-CoV-2: Severe acute respiratory syndrome coronavirus type 2
SNI: Severe neurological impairment
