# Supplementary material for: Clinical recommendations for the inpatient management of lower respiratory tract infections in children and adolescents with severe neurological impairment in Germany—2026 Update
Source: Eur J Pediatr. 2026 Nov 9;185(12):925. doi: 10.1007/s00431-026-07482-5 (PMC13748045; doi:10.1007/s00431-026-07482-5)
Supplement: Supplementary file 1 [file 431_2026_7482_MOESM1_ESM.docx]

**Clinical Recommendations for the Inpatient Management of Lower Respiratory Tract Infections in Children and Adolescents with Severe Neurological Impairment in Germany – 2026 Update**

Summary of recommendations

*This appendix was newly added in the 2026 update to provide a concise, structured overview of the clinical recommendations.*

| **Diagnostics** | **Recommendation 1.1** |  |
| --- | --- | --- |
|  | Initial diagnostic assessment of lower respiratory tract infections (LRTI) in children and adolescents with severe neurological impairment should include a complete clinical examination, assessment of vital signs, and evaluation for alternative infectious foci outside the respiratory tract. |  |
|  | **Recommendation 1.2** |  |
|  | Additional diagnostic workup should include targeted laboratory and microbiological testing, including blood cultures in severe disease, as well as collection of deep respiratory tract secretions. Colonization and infection should be critically distinguished. |  |
|  | **Recommendation 1.3** |  |
|  | Imaging should be used according to clinical indication, with lung ultrasound preferred. Chest radiography or computed tomography should be reserved for selected questions with therapeutic implications. |  |
| **Therapy** | **Recommendation 2.1** |  |
|  | Basic treatment should include consistent symptomatic care with individualized hydration and fever control, oxygen therapy in case of hypoxemia, and secretion management with inhalation, physiotherapeutic, and device-assisted respiratory support. In patients with respiratory insufficiency, early interdisciplinary assessment should be obtained. |  |
|  | **Recommendation 2.2** |  |
|  | In suspected viral LRTI, management should focus on targeted antiviral treatment where indicated, for example in influenza, and close clinical reassessment, particularly to detect bacterial superinfection or coinfection in case of clinical deterioration. |  |
|  | **Recommendation 2.3** |  |
|  | In suspected bacterial LRTI, initial empiric antibiotic therapy (ABT) should follow a pragmatic risk stratification:  – Group I: community-acquired LRTI or aspiration pneumonia: treatment with an aminopenicillin plus a beta-lactamase inhibitor.  – Group II: chronic lung disease or tracheostomized patients with recurrent LRTI: treatment with piperacillin-tazobactam. |  |
|  | **Recommendation 2.4** |  |
|  | Known *Pseudomonas* spp. colonization justifies initial broadening of the antimicrobial spectrum only in patients with a corresponding history or critical illness. In the absence of such a history and in clinically stable patients with community-acquired LRTI or aspiration pneumonia, an initial treatment attempt with an aminopenicillin plus a beta-lactamase inhibitor remains reasonable. |  |
|  | **Recommendation 2.5** |  |
|  | In patients colonized with multidrug-resistant organisms (MDRO) but without critical illness, previous microbiological findings should be considered in context. Temporary combination therapy may be considered to bridge the period until current susceptibility results are available appears preferable to primary use of reserve antibiotics. |  |
|  | **Recommendation 2.6** |  |
|  | In critically ill patients with known colonization with MDRO, initial ABT should be broadened according to the most recent susceptibility profile, followed by consistent reassessment and narrowing of therapy as part of an early antibiotic timeout. |  |
| **Secretion management and respiratory support** | **Recommendation 3.1** |  |
|  | In patients with signs of increased secretion burden or ineffective cough, established secretion management should be intensified through targeted respiratory physiotherapy, inhalation, and mechanical insufflation-exsufflation, guided by the clinical course and coordinated within an interdisciplinary team. |  |
|  | **Recommendation 3.2** |  |
|  | In patients without established secretion management, targeted respiratory physiotherapy, inhalation treatment, and mechanical insufflation-exsufflation may be initiated during the acute pulmonary exacerbation |  |
|  | **Recommendation 3.3** |  |
|  | In patients with pre-existing respiratory insufficiency, established respiratory support should be continued during LRTI and individually adjusted in case of clinical deterioration. Additional oxygen should be administered under controlled conditions with attention to ventilation, to avoid masking hypercapnic respiratory failure. |  |
| **Treatment monitoring and adjustment** | **Recommendation 4.1** |  |
|  | After 48–72 hours of ABT, a structured antibiotic timeout should be performed to jointly reassess the indication for ABT, clinical course, and treatment response - taking into account all available clinical, laboratory, microbiological, virological, and imaging findings, ideally supported by an infectious diseases or antimicrobial stewardship consultation. |  |
|  | **Recommendation 4.2** |  |
|  | If bacterial infection is unlikely, ABT should be discontinued. If bacterial infection remains possible or likely and the patient is clinically improving, the antimicrobial spectrum of the ABT should be narrowed and, where possible, treatment should be switched to oral or enteral step-down therapy. |  |
|  | **Recommendation 4.3** |  |
|  | In case of absent clinical improvement or deterioration, a comprehensive reassessment should be performed. This includes optimization of supportive care, critical review of alternative diagnoses, completion of pathogen-directed diagnostics, and targeted adjustment of the ABT, taking microbiological results, susceptibility testing, and individual risk factors into account. |  |
|  | **Recommendation 4.4** |  |
|  | In Group I patients (initially treated with an aminopenicillin plus a beta-lactamase inhibitor), lack of response in the absence of pathogen detection should prompt empiric broadening by switching to piperacillin-tazobactam. |  |
|  | **Recommendation 4.5** |  |
|  | In Group II patients, initially treated with piperacillin-tazobactam, lack of response in the absence of pathogen detection should prompt individualized escalation, for example by adding tobramycin or fosfomycin, or by switching to alternative regimens such as cefepime-based combinations, levofloxacin, or meropenem. |  |
| **Duration of therapy** | **Recommendation 5.1** | |
|  | The duration of ABT should be guided by the clinical course and is usually (5–)7 days in uncomplicated LRTI, with longer treatment required in complicated pneumonia or bronchiectasis. Oral step-down therapy should be guided by susceptibility results. | |
|  | **Recommendation 5.2** | |
|  | Discharge may be considered in patients with stable general condition, individually improved work of breathing, and safe respiratory and cough support, including moderately intensified support, provided that a documented de-escalation and follow-up plan is available. | |
|  | **Recommendation 5.3** | |
|  | In patients with recurrent difficult-to-treat bacterial LRTI, advance planning of empiric ABT should be pursued. In patients repeatedly requiring intravenous treatment, long-term venous access may be considered. | |
| **Prevention and prophylaxis** | **Recommendation 6.1** | |
|  | Preventive measures include consistent basic hygiene, hygienic device management and ventilation care, optimization of ventilation, reflux, and seizure control, as well as regular oral and dental care to reduce the risk of aspiration. | |
|  | **Recommendation 6.2** | |
|  | In patients with recurrent bacterial LRTI caused by Gram-negative pathogens, inhaled antibiotic prophylaxis or eradication strategies may be considered. There is no robust evidence for enteral antibiotic prophylaxis. | |
|  | **Recommendation 6.3** | |
|  | Immunization should be completed according to national recommendations, including RSV, influenza, COVID-19, and pneumococcal immunization. | |
| **Palliative care and ethical aspects** | **Recommendation 7.1** | |
|  | The treatment of children and adolescents with severe neurological impairment aims, irrespective of acute infections, to relieve symptoms and maintain quality of life. In patients with complex or progressive symptom burden, pediatric palliative care should be involved early. | |
|  | **Recommendation 7.2** | |
|  | Treatment decisions should be based on medical indication and the patient’s will or presumed will and should be made through shared decision-making with patients, legal guardians, and interdisciplinary teams. Relief of distressing symptoms should remain a priority irrespective of the overall treatment goal. | |
|  | **Recommendation 7.3** | |
|  | In life-limiting conditions, limitations of therapy should be considered if intensive care measures are highly likely to impose a disproportionate burden or only to prolong the dying phase. | |
|  | **Recommendation 7.4** | |
|  | Structured advance care planning, including written guidance for emergency situations, should be pursued for all patients with SNI and regularly adapted to the current clinical and life situation. | |
